# Palliative care in the Eastern Mediterranean: comparative analysis using specific indicators

**DOI:** 10.1186/s12904-022-01047-7

**Published:** 2022-10-03

**Authors:** Miguel Antonio Sánchez-Cárdenas, Nasim Pourghazian, Eduardo Garralda, Danny van Steijn, Slim Slama, Edgar Benítez, Marie-Charlotte Bouësseau, Carlos Centeno

**Affiliations:** 1 ATLANTES Global Palliative Care Observatory, Institute for Culture and Society, University of Navarra, 31080 Pamplona, Spain; 2 IdiSNA—Instituto de Investigación Sanitaria de Navarra, Pamplona, Spain; 3 NCD Prevention (NCP) Unit in the Department of UHC/NCDs at the World Health Organizations Regional Office for the Eastern Mediterranean (WHO EMRO), Cairo, 11371 Egypt; 4 DATAI, Institute of Data Science and Artificial Intelligence, University of Navarra, 31080 Pamplona, Spain; 5 University of Navarra (UNAV), TECNUN School of Engineering, San Sebastián, Spain; 6 Integrated Health Services, World Health Organisation, CH1211 Geneva, Switzerland

**Keywords:** Palliative care, Development, Specialized services, Provision, Public health, Eastern Mediterranean, National-level

## Abstract

**Background:**

Monitoring the development of palliative care (PC) illustrates the capacity of health systems to respond to the needs of people experiencing serious health-related suffering.

**Aim:**

To analyse comparatively the situation of PC in the countries of the Easter Mediterranean region using context-specific indicators.

**Method:**

An online questionnaire with 15 context-specific PC indicators investigating service provision, use of medicines, policy, education, and vitality was designed. Authors Institution 1 nominated in-country experts to complete the survey. Data were analysed using a comparative description of indicators per domain and a multivariate analysis.

**Results:**

In-country experts were identified in 17/22 countries. 12/17 contributed to the survey. In total, 117 specialized PC services were identified. Specialized services per population ranges from 0.09 per 100,000 inhabitants in Lebanon and Saudi Arabia, Qatar and Kuwait; to zero services in the Occupied Palestinian Territories. On average, opioid consumption was 2.40 mg/capita/year. National PC strategies were reported in nine countries. In six countries, PC is officially accredited either as a specialty or sub-specialty, and PC mandatory courses are implemented in 36% of medical schools and 46% of nursing schools. National PC associations were documented in six countries. A higher pattern of development was identified in Jordan, Kuwait, Saudi Arabia, Oman, Lebanon, Qatar.

**Conclusions:**

Despite a higher development in the Arabian Peninsula, the region is characterised by a very low provision of specialized PC services and opioid consumption. Policy improvements represent an opportunity to improve access to PC.

**Supplementary Information:**

The online version contains supplementary material available at 10.1186/s12904-022-01047-7.

## Background

The Eastern Mediterranean region of the World Health Organization (EMRO) comprises 22 member states and territories and a population of approximately 679 million people. In the Eastern Mediterranean Region (EMR), various socio-health contexts influence the development of palliative care [1]. In 2016, 4,138,382 deaths were registered in the region, 43% of which were related to conditions suitable for palliative care. Cancer and Cardiovascular disease were the two main causes of serious health-related suffering [2].

To date, some investigations have worked under the assumption that for national health systems to respond adequately to suffering associated with advanced disease, it is necessary to estimate health system’s capacity to match the needs of patients, caregivers and families. In 2017, a first cross-sectional evaluation of palliative care activity in EMR was led by Balsam Lebanon palliative care center. It identified an incipient Palliative Care activity with a scarce number of specialized palliative care services and very low consumption of medicines for pain relief [3]. Contemporary studies further pointed out barriers related to the absence of palliative care national policies; gaps in access to essential analgesics, insufficient palliative care education for health professionals and volunteers, low public awareness, and a lack of collaborative work in scientific associations [4, 5]. Indeed, according to the global map, the majority of the region (18/22 countries, 82%) had isolated or non-existent palliative care provision, meaning little and narrowly distributed services across territories, and an occasional morphine availability for pain relief (if existing) [6].

In 2019, a network of palliative care experts in the EMR was initiated to promote a regional roadmap to understand the needs and the palliative care development and integration in national health systems. The aim of this study is to analyse comparatively the situation of Palliative Care in the countries of the Eastern Mediterranean region using context-specific indicators.

## Methods

### Indicators selection

Context-specific indicators were agreed through a two-round Delphi consensus process by the experts’ network. It was promoted by the authors institute 1 and coordinated authors institute 2. A total of 15 indicators depicting the four domains described in the Public Health Strategy for Palliative Care integration: policy, education, use of medicines, and service provision; were covered (ref. Stjnsward). Additionally, one extra domain was explored: Palliative Care vitality. All 15 were agreed as feasible and relevant indicators (Table 1). Details of this process are presented in a separate publication.

**Table 1:** Selected indicators to evaluate the development of palliative care in EMR^a^

Indicators of palliative care	
1. Existence of a current national palliative care plan, programme, policy or strategy.	
2. Number of specialized palliative care services in the country per population.	
3. Paediatric palliative care provision.	
4. Line item for palliative care in the national health budget for the Ministry of Health or equivalent government agency.	
5. Education for pre-qualification doctors/nurses.	
6. Availability of morphine and other strong opioids.	
7. Inclusion of palliative care services in the basic package of health services.	
8. Existence of professional vitality regarding palliative care^b^.	
9. Reported annual opioid consumption- excluding methadone -in morphine equivalence (ME) per capita.	
10. Existence of a specific palliative care national law.	
11. Level of public awareness of palliative care.	
12. Existence of a process of official specialisation in palliative medicine for physicians, recognised by the competent authority.	
13. Palliative care inclusion in health insurance plans.	
14. Availability of centres of excellence for palliative clinical care, education and research.	
15. Existence of grants to finance palliative care research.	

^a^Eastern Mediterranean region

^b^‘palliative care vitality’ indicator characterizes the professional infrastructure for developing the palliative care field over time. This indicator explores aspects regarding professional vitality in PC, such as the existence of at least one national PC association, a PC services directory, a national journal of PC, and the celebration of PC congresses

### On-line survey

An online survey consisting in 27 questions exploring the 14 indicators was designed and piloted (The indicator on opioid consumption was obtained from the global reports by the IU Walther Center in Global Palliative Care & Supportive Oncology [7]). The type of questions used differed: 11 open questions, 11 ordinal questions (Yes/No/In progress or a scale), and five nominal questions (supplementary material 1). The survey was sent by August 2020 via email to the nominated national in-country experts. Experts were encouraged to collaborate with official sources of information (e.g. Ministries of Health or Government organizations) to obtain the information.

### Source of information: network of in-country experts

The Eastern Mediterranean region of the World Health Organization nominated in-country experts as valid and independent sources to report estimations on the national Palliative Care situation. National Ministry of Health endorsement and personal written agreement of the expert was required. Main selection criteria were their knowledge on Palliative Care in their respective countries, previous participation in other studies, and scientific publications relevant to the topic of study.

### Data analysis

Simple descriptive measures were used to present information comparatively per component: health policy, service provision, Palliative Care education, opioids consumption and Palliative Care vitality.

#### Multivariate analysis

In order to synthesize indicators, all variables were converted to a binomial scale, based on their respective median values, and grouped according to the dimensions evaluated: politics, education, medical, services, and vitality. All variables were considered regardless of whether they had missing data. The final value of each dimension corresponded to its average, which was later transformed to the respective rank for each country in each dimension. As they are ordinal variables, a polychoric correlation matrix was calculated to avoid underestimation of the relationship, if the Pearson statistic was used. The significance of the relationship was tested by means of the likelihood ratio (LR), with *p* < 0.05. Finally, for the ranked values of each dimension for each country, a matrix of distances was generated from their medians, which allowed the creation of a dendrogram where the proximity of the countries can be seen in a multivariate manner.

## Results

In-country experts were identified in 17/22 countries (21 Member States and the Occupied Palestinian territories). Responses were obtained from 12 of them: Egypt, Iran, Iraq, Jordan, Kuwait, Lebanon, Morocco^1^, Occupied Palestinian Territories, Oman, Pakistan, Qatar, and Saudi Arabia. This sample represents 55% of the countries in the region, and 71% of the countries where an in-country Palliative Care expert was identified (see Table 2 for Informants’ background and expertise). The experts identified from Afghanistan, Tunisia, United Arab Emirates, and Sudan, did not participate in the consensus and no expert was identified in five countries of the region: Bahrain, Djibouti, Syrian Arab Republic, Somalia and Yemen.

**Table 2:** Informants’ background and expertise

Country	Informant	Background	Participation in relevant publications on palliative care development
Egypt	Samy Alsirafy	Palliative Medicine Unit (Kasr Al-Ainy Center of Clinical Oncolgy & Nuclear Medicine). Established the first university hospital-based PC unit in Egypt in 2008.	-Osman et al. *Atlas of Palliative Care in the Eastern Mediterranean Region 2017*. -Alsirafy SA, et al. *Preferred Place of Death for Patients With Incurable Cancer and Their Family Caregivers in Egypt. AJHPM*. 2019;36(5):423–428
Iran	Maryam Rassouli	Shahid Beheshti University of Medical Sciences, Tehran. Specialized in PC, has research interests in PC.	-Osman et al. *Atlas of Palliative Care in the Eastern Mediterranean Region 2017* -Barasteh S, Rassouli M, et al. *Palliative Care in the Health System of Iran: A Review of the Present Status and the Future*. APJCP. March 2020 Challenges
Iraq	Samaher A. Fadhil	Affiliated to the Children welfare Teaching Hospital, Pediatric oncology center, Baghdad Medical City; has research interests in PC.	-Fadhil S.A., Ghali H.H. (2021) *The Current Situation of Palliative Care Services in Iraq. In: Silbermann M. (eds) Palliative Care for Chronic Cancer Patients in the Community*. Springer, Cham -Fadhil I, Lyons G, Payne S*. Barriers to, and opportunities for, palliative care development in the Eastern Mediterranean Region*. Lancet Oncol. 2017;18(3)
Jordan	Omar Shamieh	Chairman of the PC department at King Hussein Cancer Center since 2011, led the establishment of the National Palliative & Home Care Strategic Framework.	-Shamieh O, et al. *Gaining Palliative Medicine Subspecialty Recognition and Fellowship Accreditation in Jordan*. JPSM, Vol. 60, 5, 2020, pp. 1003–1011.
Kuwait	Iman Al Diri	Expert in cancer pain management at the Kuwait Cancer Control Center and co-author of several PC-related publications.	-Osman et al. PALLIATIVE CARE. In book: *Cancer Control: Eastern Mediterranean Region Special Report* _new Publication (pp.82) -Sánchez-Cardenas M. et al. *Region-specific macroindicators on palliative care development in the Eastern Mediterranean Region: a Delphi study.* EMJH, 2022
Lebanon	Hibah Osman	Founder of Balsam – Lebanese Center for PC, and of the PC Program at the American University of Beirut	-Osman et al. *Atlas of Palliative Care in the Eastern Mediterranean Region 2017*. -Osman H, Ramia JA. *Assessing physicians’ perception of home based palliative care services in the beirut area*. BMJ Support Palliat Care. 2015 Apr; 5 Suppl 1:A28
	Huda Abu-Saad Huijer	Specialist in PC at the School of Nursing, American University of Beirut. Research focused primarily on pain management and PC in children and adults.	-Abu-Saad Huijer, H. *Evidence-Based Palliative Care Across the Life Span*. John Wiley & Sons; 2001. -Abu-Saad Huijer, H, et al. *A Mapping of Nursing and Midwifery Research in the Eastern Mediterranean Region* (EMR). *EMHJ*. 24(7): 640–650
	Myrna A. A. Doumit	Alice ramez Chagoury School of Nursing, LAU and Lebanese American University since Feb. 2011. Member of the national committees of PC and Pain under MoH.	-Doumit MAA, et al. *The lived experience of Lebanese oncology patients receiving palliative care*. Eur J Oncol Nure 2007 Sep;11(4):309–19 -Doumit, M**.** (2011). *Lebanon - making a start in palliative care*. Hospice Information Bulletin, 8, 2, 10.
Morocco	Asmaa El Azhari	Responsible for the PC Unit of Mohammed VI Center for Cancer Treatment, Ibn Rochd Casablanca. Interests in PC for oncological patients.	-Sánchez-Cardenas M. et al. Region-specific macroindicators on palliative care development in the Eastern Mediterranean Region: a Delphi study. EMJH, 2022
Oman	Bassim Al Bahrani	Consultant Medical Oncologist at National Oncology Centre, Royal Hospital, and chief operating officer at the Gulf International Cancer Center. Member of many oncology associations.	-Osman et al. *Atlas of Palliative Care in the Eastern Mediterranean Region 2017*, -Al Bahrani B, Mehdi I. *The Need for Regulatory Reforms in the Use of Opioids for Pain*. May 2018. Gulf journal of oncology, 1(27):52–59.
Pakistan	Muhammad Atif Waqar	Assistant Professor and the founding chief of the Section of Palliative Medicine in the Dept of Oncology at the Aga Khan University, Karachi. Set up the city’s first multidisciplinary PC service.	-Waqar MA, et al. Provision of Palliative Care for Oncological Patients in Pakistan: A Review of Challenges and Current Practices. January 2021. In book: Palliative Care for Chronic Cancer Patients in the Community (pp.479–486)
Occupied Palestinian Territory^a^	Hani S. Ayyash	Specialized in clinical Hematology at the European Gaza Hospital, has research interest in PC.	-Osman et al. *Atlas of Palliative Care in the Eastern Mediterranean Region 2017*. -Bar-Sela G, et al. *Training for awareness of one’s own spirituality: a key factor in overcoming barriers to the provision of spiritual care to advanced cancer patients by doctors and nurses*. Palliat Support Care. 2019;17(3):345–352
Qatar	Azza Adel Ibrahim Hassan	American Board of Hospice and Palliative Medicine since 2003, and current Program Director of Supportive and PC Unit at the National Center for Cancer Care and Research-Hamad Medical Corporation	-Silbermann M, (…), Hassan AAI. *The Middle East Cancer Consortium promotes palliative care*. Lancet (London, England). 2015 -Kreuter M. et al. *Palliative care in interstitial lung disease: living well*. Lancet Respir Med. 2017
Saudi Arabia	Sami Ayed Alshammary	Works at the Department of PC, Comprehensive Cancer Center, King Fahad Medical City, MoH, since 2010. Interested in symptom management and competences in PC for physicians.	-Alshammary SA, et al. Palliative care in Saudi Arabia: Two decades of progress and going strong. Journal of Health Specialties 2 (2), 59. -Alshammary SA, et al. *Development of Palliative and End of Life Care: The Current Situation in Saudi Arabia’*. Cureus. 2019 Mar; 11(3): e4319

^a^One respondent from the Occupied Palestinian Territory wished to remain confidential

### Palliative care specialized services

**Fig. 1:**
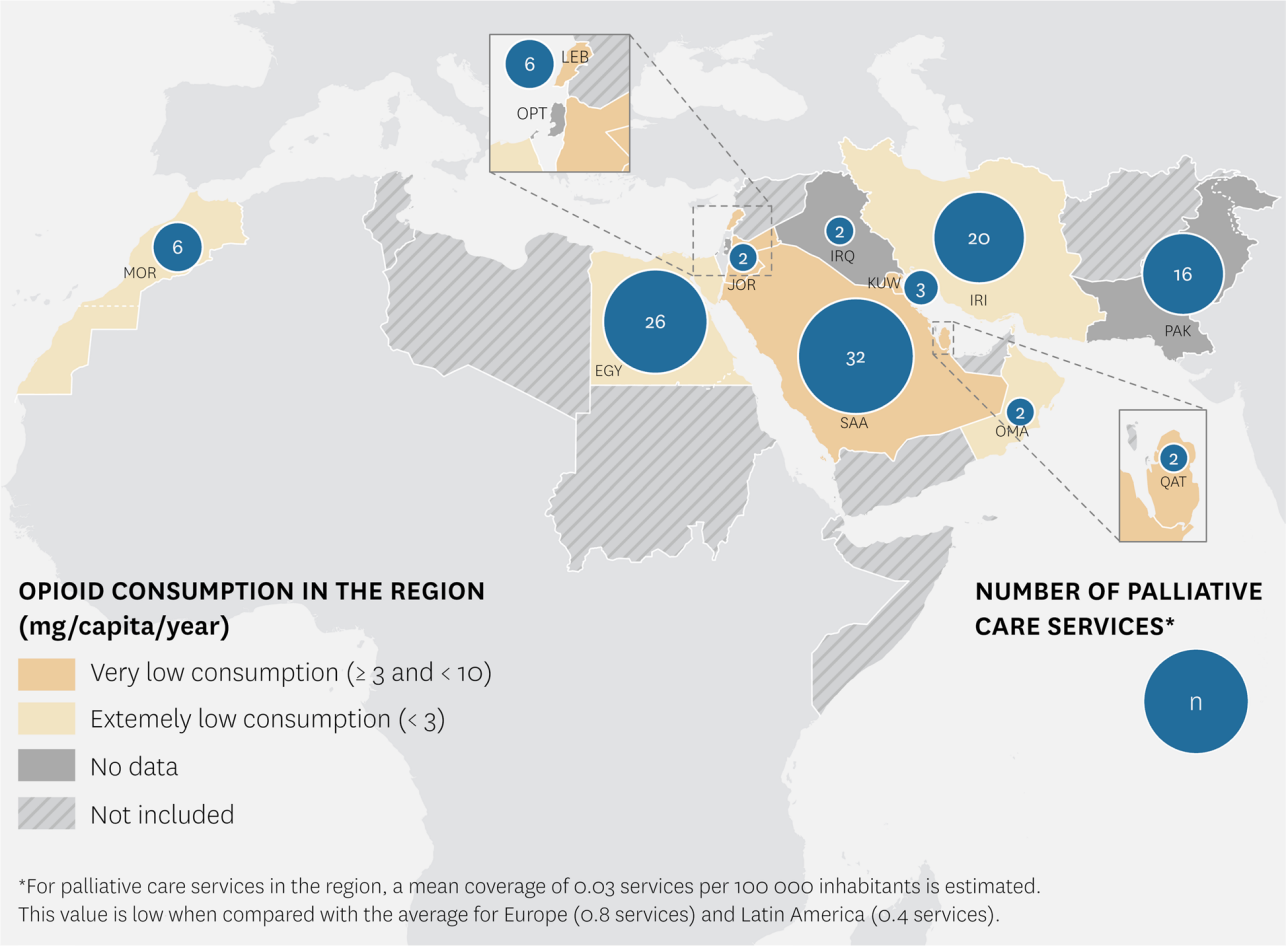
Number of palliative care services and opioid consumption in EMR

Across the region, 117 specialized Palliative Care services were identified (see Fig. 1). The highest reports correspond to Egypt and Saudi Arabia. Specialized services per population ranges from 0.09 per 100,000 inhabitants in Lebanon and Saudi Arabia, Qatar and Kuwait; to zero services in the Occupied Palestinian Territories. On average, services per population across evaluated countries is 0.05 ± 0.03. Countries provide Palliative Care through specialized hospital support teams and inpatient units (*n* = 9), home care programs (*n* = 9), outpatient services (*n* = 8), and hospice-type resources (*n* = 5). Some paediatric Palliative Care services are available in Kuwait and Qatar. Isolated paediatric provision was reported in Egypt, Jordan, Lebanon, Morocco, Pakistan, Occupied Palestinian Territories, and Saudi Arabia.

Geographic spread of services throughout the national territories was estimated in a 1–10 scale, 10 being the highest distribution. Saudi Arabia and Jordan reported the best mean dispersion values: 5.6 and 4.2, respectively. Mean spread for the diverse types of services varies: home care programs (4.30 ± 2.22), outpatient Palliative Care services (3.91 ± 2.34), hospital support teams (3.28 ± 2), and hospices (2.03 ± 1). Some centres of excellence have been documented in Jordan, Morocco, Oman, Pakistan and Saudi Arabia. They supply an exceptionally high concentration of expertise and related resources centered on Palliative Care, including a significant education, research, and clinical capacity building to ensure that they understand and can relate to patients.

### Opioid consumption

Sixteen countries in the region reported in 2017 an average opioid consumption rate of 2.40 mg/capita/year (in equivalents of oral morphine). Significant differences exist between countries like Saudi Arabia, Bahrain and Kuwait, that informed values between 5.81 and 7.20 mg per capita, and a greater number of countries reporting a null consumption (see Fig. 1).

Six countries report having both oral and injectable morphine available at all times: Jordan, Kuwait, Qatar, Saudi Arabia, Oman, and Palestine. Other countries report a rather inconsistent availability, overall regarding injectable morphine: Egypt, Morocco or Pakistan, where injectable morphine is occasionally available. All countries report consistent availability of medicines on the first rung of the WHO analgesic ladder, five report tramadol and codeine available at all times, and three report consistent availability of fentanyl.

#### Health policy favouring palliative care

Seven countries reported a specific national strategy or plan for Palliative Care: Iran, Jordan, Kuwait, Lebanon, Oman, Qatar and Saudi Arabia. In Kuwait, Qatar, and Saudi Arabia, the strategies have been implemented and are periodically evaluated. Egypt, Morocco, and Pakistan informed that their national cancer strategies include references to Palliative Care. Some countries like Iran, Jordan, Kuwait, Lebanon, Morocco, Qatar and Saudi Arabia have placed a designated staff person responsible for Palliative Care within the Ministry of Health (or equivalent body) (Table 3). National regulations related to Do Not Attempt Resuscitation were reported in five countries. In six countries Palliative Care services are part of the health benefits plan, although in Qatar this only applies to cancer patients and older persons.

**Table 3:** Indicators on Health policy related palliative care in EMR

Country	National strategy		PC Law	DNAR^**a**^ related regulations	PC in basic package	Funding for palliative care				
	PC in cancer	PC specific				Gov Reg.^**b**^	Gov Irreg^**c**^	NGOs^**d**^	Private insur.^**e**^	Direct paymt.^**f**^
**Egypt**	**√**	x	x	x	x	**√**	x	x	x	x
**Iran**	x	**√**	x	x	x	x	x	**√**	x	x
**Iraq**	x	x	x	x	x	x	**√**	x	**√**	x
**Jordan**	x	**√**	x	**√**	**√**	x	x	x	**√**	x
**Kuwait**	x	**√**	x	x	**√**	x	**√**	x	x	x
**Lebanon**	x	**√**	IP	**√**	**√**	**√**	x	x	**√**	x
**Morocco**	**√**	x	x	x	x	x	x	x	x	**√**
**Oman**	IP	x	x	**√**	**√**	**√**	x	x	**√**	x
**Pakistan**	**√**	x	x	x	x	x	x	x	**√**	**√**
**Palestine**	x	x	x	x	x	x	x	x	x	**√**
**Qatar**	x	**√**	√	**√**	**√**	**√**	x	x	**√**	x
**Saudi Arabia**	x	**√**	IP	**√**	**√**	**√**	x	x	**√**	x

*IP* In progress

^a^*DNAR* Do not attempt resuscitation

^b^Government-funded on a regular basis

^c^Government-funds on irregular base

^d^Non-profit Organisations

^e^Private health insurance

^f^Direct payment by patients

Palliative Care funding is reported through line items in national health budgets for Egypt, Jordan, Kuwait, Lebanon, Oman, Qatar and Saudi Arabia; while for others funding comes from private health insurance and direct payments to health centres or non-governmental organizations (Iran, Iraq or Pakistan). Some countries do not have any funding available and allocated for Palliative Care, such as Morocco or the Occupied Palestinian Territories.

### Palliative care education

The existence of palliative medicine official specialization programs was differently reported in six countries. Lebanon recognizes Palliative Care as a specialty; Qatar, Jordan, Saudi Arabia, and Iran as a sub-specialty; and, in Pakistan, Palliative Care it is accredited as a special area of competence. Iraq, Kuwait and the Occupied Palestinian Territories officially recognize specializations completed abroad, a specialization program is in process in Oman, and in Morocco, informal accredited trainings are available. The estimated number of palliative medicine specialists varied across countries: around 50 in Qatar and Saudi Arabia, 15 in Iran, and four in Pakistan. In Iraq, three specialists are recognized as such having specialized abroad (Table 4).

**Table 4:** Indicators on education and training for palliative care in EMR

Country	% medical schools with mandatory course	% nursing schools with mandatory course	Accreditation in palliative medicine
Lebanon	100%	100%	Specialty
Qatar	100%	100%	Sub-speciality
Kuwait	100%	100%	Abroad specialization recognized
Oman	50%	100%	Specialization process in progress
Jordan	33%	67%	Sub-speciality
Saudi Arabia	11%	17%	Sub-speciality
Morocco	70%	0%	Informal process of training is available
Palestine	50%	0%	Official recognition of specialisation done abroad recognized
Pakistan	9%	4%	Special area of competence or another advanced accreditation diploma
Egypt	3%	0%	Informal training available
Iran	0%	0%	Sub-speciality
Iraq	0%	0%	Official recognition of specialisation done abroad

In Kuwait, Lebanon and Qatar all medical schools offer Palliative Care as a mandatory separate subject. In Morocco, Oman, Palestine and Jordan Palliative Care is also offered at a lesser proportion (30–70% of the medical faculties). Some countries teach Palliative Care as part of a related course (e.g. oncology); for example, Iran and Saudi Arabia; some others offer Palliative Care as a transversal element of medical training integrated into other subjects: Morocco, Pakistan, Jordan, Qatar, and Kuwait. In Kuwait, Lebanon, Qatar and Oman Palliative Care training is included in all nursing degrees. In Jordan, 67% of nursing programs do include Palliative Care; in Iran or Saudi Arabia, nursing programs do cover Palliative Care under oncology nursing, home care, and pain management (at a 100 and 50% of nursing schools, respectively).

### Palliative care vitality

Various signs of “Palliative Care vitality” were identified. Professional cooperation with specialties outside Palliative Care was reported in eight countries, policy and professional meetings took place in seven countries; clinical guidelines or standards were published in six countries; national conferences were held in five states. Furthermore, Jordan, Kuwait, Lebanon, Morocco, Oman, and Saudi Arabia have a national Palliative Care scientific association. Four states published a national Palliative Care directory of resources (Kuwait, Saudi Arabia, Morocco and Qatar). Although no country reported specialized Palliative Care publications, Jordan and Qatar report availability of grants to fund Palliative Care research. Public awareness of Palliative Care is perceived differently across countries (Table 5).

**Table 5:** Indicators on vitality^a^ in palliative care at EMR

Country	National association	National journal	Services Directory	Nat. conference	Clinical standards	Profess.or policy meetings	Profess. coop.	Research funding	Public awareness ^**b**^
**Jordan**	**√**	X	IP	**√**	**√**	**√**	**√**	Many	Medium
**Qatar**	X	X	**√**	IP	**√**	**√**	**√**	Many	High
**Kuwait**	**√**	X	**√**	IP	**√**	**√**	**√**	NA	Medium
**Saudi Arabia**	**√**	IP	**√**	√	**√**	**√**	IP	Some	Medium
**Lebanon**	**√**	X	IP	**√**	IP	**√**	**√**	Almost none	Medium
**Morocco**	**√**	X	**√**	X	**√**	IP	**√**	Some	Significant
**Iran**	X	X	X	**√**	IP	**√**	**√**	Some	Little
**Pakistan**	IP	X	X	**√**	IP	IP	**√**	None	Medium
**Oman**	**√**	X	X	X	√	√	X	Almost none	Medium
**Egypt**	X	X	X	X	X	X	IP	Almost none	Medium
**Iraq**	X	X	X	X	X	IP	**√**	Almost none	Little
**Palestine**	X	X	X	X	X	IP	X	None	Little

*IP* In progress, *NA* No information available

^a^Understood as the existence of professional scientific activities or evidence of public awareness in the country

^b^Little: Most people don’t care about palliative care; Medium: Some sectors; Significant: A significant proportion; High: Generalised in the society

### Sub-regional patterns of development

As a result of the binomial classification, Kuwait, Oman, Qatar, Saudi Arabia and Jordan reached the highest ranked values in the availability of resources to develop Palliative Care programs (supplementary material 2). The indicators summarized by each dimension and classified in ranks in the group of countries (Table 6), showed, in general, two groups of countries: Oman, Lebanon, Qatar, Jordan, Kuwait, Saudi Arabia, and Palestine, with upper values; and Iran, Morocco, Iraq, Egypt, and Pakistan, with lower. Within the group of low values, a similar profile is observed for Iran and Morocco, although Morocco presents an unusually high position for the variable Vitality compared to Iran and its group. In that same group, Egypt and Pakistan show similarities, however, Pakistan ranks higher for Education and Vitality. A particular behaviour is presented by Palestine, which is consistently located in the last ranks except in the Medicines indicator or Iraq, with low positions across all dimensions. Within the upper cluster, Jordan, Kuwait and Saudi Arabia reported the highest positions in all indicators. Qatar would follow with high values in almost all indicators except Services, and, Oman and Lebanon, are grouped presenting high values for the Politics and Education indices.

**Table 6:** Ranking of overall performance of PC indicators in EMR

Country	Services	Medicines	Education	Policies	Vitality	Score^**a**^
Qatar	6	10.5	11.5	12	10.5	50.5
Saudi Arabia	12	10.5	5	10.5	10.5	48.5
Kuwait	9.5	10.5	9	9	8	46
Jordan	9.5	7	9	7.5	10.5	43.5
Lebanon	6	7	11.5	10.5	6.5	41.5
Oman	4	7	9	7.5	4.5	32
Morocco	6	4	5	2.5	10.5	28
Pakistan	9.5	2	5	4.5	4.5	25.5
Egypt	9.5	2	1.5	6	1.5	20.5
Iran	3	5	5	2.5	6.5	22
Palestine	2	10.5	5	1	1.5	20
Iraq	1	2	1.5	4.5	3	12

^a^Arithmetic sum of the scores obtained in each dimension by country

## Discussion

Palliative care development in EMR seems geographically heterogeneous and inconsistent across components, with some common characteristics among the countries of the region. About half the countries observed have developed national Palliative Care strategies, have included Palliative Care in the regulatory system or allocated of funds for Palliative Care. These policy resources, typically, correspond to countries where Palliative Care access and provision is generalized. However, there are very few specialized services on average (0.03 services per 100,000 inhabitants), especially if compared with other world regions’ average (0.81 in Europe, 0.43 in Latin America). Even considering best provided countries (Lebanon: 0.09; Saudi Arabia: 0.09), ratios stand away from, for instance, the EAPC standards, that suggest 2 services per 100,000 inhabitants. Subsequently, geographic spread of services remains scarce meaning that population needs, wherever they live, are uncovered.

Opioid consumption is extremely low if compared to international recommendations and averages, and uneven across countries. On average, the region distributes 2.40 mg/capita/year, versus the 200 mg/capita/year that has been lately estimated to be an adequate use of medicines [8] or the 107 mg/capita/year reported for 49 countries of WHO Europe [9]. Besides, within the region, flagrant differences exist between data from Saudi Arabia, Bahrain or Kuwait (5.81–7.20 mg/capita), and a number of countries reporting a null consumption. Gaps in access to essential medicines to relieve pain, as largely pointed out in the literature, still is one of the greatest barriers to relieve SHS in the EMR [4].

On the contrary, some findings allow some optimism, particularly if compared to results published in the EMR 2017 Atlas of Palliative Care [3], even though there are methodological differences. There is incipient evidence on an increase in specialized Palliative Care services in Saudi Arabia, Qatar, Egypt, Iran and Pakistan. The number of countries recognizing palliative medicine as an official accredited specialization has also increased from four countries to six, and the proportion of medical and nursing programs teaching Palliative Care compulsorily to future professionals has grown. Furthermore, regarding health policies, Qatar has implemented a Palliative Care law that was initiated in 2017, and stand-alone national Palliative Care strategies are available in Saudi Arabia, Kuwait, Qatar, Iran, Lebanon and Jordan (in 2017 just Tunisia reported one). Some vitality signs such as the settlement of national Palliative Care professional associations or the cooperation with other specialities in a number of countries suggest that the discipline is ingraining and moving forward. The very EMR Palliative Care expert’s network is a valuable symptom.

These findings, generally, echo studies that have explored barriers to the development of Palliative Care in the region [4, 6, 10, 11]. Worryingly, the opioid consumption, away from international standards [8], put all countries at very low levels of consumption (3–10 mg/capita) or even at extremely low levels (< 3 mg/capita). This means a continuation on previous analyses that claimed equitable access to medicines for pain control was problematic in EMR [12–14]. A further barrier to take into account includes the great suffering associated with humanitarian emergencies and crises, a reality for a number of countries (or regions) within the region: Iraq, Syria, Yemen, Sudan, and the Occupied Palestinian Territories. Also, countries that receive massive migrations from conflict areas -such as Lebanon for instance- face a situation where their health systems are having a greater demand. To develop strategies in accordance to sociosanitary conditions is needed, where a useful lever to improve palliative care provision is the integration of this care into the primary care level [15].

Differently to past studies, this regional diagnosis of the palliative care development at EMR has identified different sub-regional patterns. The development of palliative care is noticeably higher in countries of the Arabian Peninsula (especially in its northern and central areas: Jordan, Saudi Arabia and Kuwait). The identification of these differentiated groups demands a sub-regional customized design of regional health strategies according to the resources and needs of each country. This should allow to promote the inclusion of palliative care in the health benefit package for all patients, its inclusion in national health systems or private health insurances, to establish a national palliative care strategy with an operational implementation framework where inexistent; to increase the palliative care services (especially home, outpatient and paediatric ones), to establish a strategy towards the use of pain medicines for symptom relief, and the design of a general understanding strategy around advanced disease and the role of palliative care throughout life and as a component of Universal Health Coverage.

The methodology used in this study represents an opportunity in itself as per the joint work between the World Health Organization and the EMR experts’ network; the use of consensus set of indicators for the region; data quality, and the support of national authorities in data collection. Nevertheless, two inherent limitations to an expert’s estimation study must be considered when interpreting results: a) the limited number of participating countries, possibly due to selection criteria of informants or the non-existence of Palliative Care activity; b) the use of new somewhat subjective indicators such as the levels of Palliative Care public awareness, existence of centres of excellence, or the availability of research grants that may favour bias in responses.

In view of these findings, future research should concentrate upon renewing data collection with this set of indicators in order to see whether structural improvements in the fields of education, policy and professional activity and advocacy, do indeed affect a positive implementation in the number of specialized services and the use of medicines for pain relief. Furthermore, given the subjectivity of the some of the indicators, future research would also benefit from a deeper exploration of the interviewees’ perceptions of challenges that must be solved in order to increase access to palliative care.

## Conclusions

There is disparity in the palliative care development within the region, making it necessary to increase health policy and strategies actions for countries with fewer resources. Both the opportunities and priority areas identified in this study. The status of development of Palliative Care in the region is characterised by low availability of specialized Palliative Care services and an infimum opioid consumption rate across countries. On the other hand, education, policy, and vitality-related areas could represent an improving trend in terms of Palliative Care structural elements that may favour future development.

### Supplementary Information

**Additional file 1.**

**Additional file 2.**

## Data Availability

Data on the development of palliative care in EMR will be available in a regional atlas, published by the NCD Prevention (NCP) Unit in the department of UHC/ NCDs at the World Health Organization Regional Office for the Eastern Mediterranean, in collaboration with the ATLANTES Global PC Observatory. To request data on this study, please email egarralda@unav.es, corresponding author of this paper.

## Appendix

### Eastern Mediterranean Regional Expert Network on Palliative Care

Egypt (Samy Alsirafy^8^, Reda S. Rizkallah^9^), Islamic Republic of Iran (Maryam Rassouli^10^), Iraq (Samaher A. Fadhil^11^), Jordan (Omar Shamieh^12^), Kuwait (Iman Al Diri^13^), Lebanon (Hibah Osman^14^, Abu-Saad Huijer^15^, and Myrna A.A. Doumit^16^), Morocco (Asmaa El Azhari^17^), Oman (Bassim Al Bahrani^18^), Pakistan (Muhammad Atif Waqar^19^), Occupied Palestinian Territory (Hani S. Ayyash^20^), Qatar (Azza Adel Ibrahim Hassan^21^), Saudi Arabia (Sami Ayed Alshammary^22^), Dubai (Mona Tareen^23^).

^8^Palliative Medicine Unit, Kasr Al-Ainy Center of Clinical Oncology & Nuclear Medicine, School of Medicine, Cairo University

^9^Children’s Cancer Hospital – Egypt

^10^Shahid Beheshti University of Medical Sciences, Tehran

^11^Children’s Welfare Teaching Hospital, Pediatric Oncology Center, Baghdad Medical City

^12^Department of Palliative Care, King Hussein Cancer Center

^13^Kuwait Cancer Control Center

^14^Balsam – Lebanese Center for Palliative Care

^15^School of Nursing, American University of Beirut

^16^Alice Ramez Chagoury School of Nursing, Lebanese American University

^17^Palliative Care Department of Mohammed VI Center for the Treatment of Cancer UHC Ibn Rochd Casablanca

^18^National Oncology Centre, Royal Hospital, Muscat

^19^The Aga Khan University, Karachi

^20^European Gaza Hospital and Confidential

^21^National Center of Cancer Care and Research-Hamad Medical Corporation

^22^King Fahad Medical City, Ministry of Health, Riyadh

^23^American Hospital Dubai

## Footnotes

1 Reported figures about Morocco were not provided nor endorsed by the Ministry of Health.
