# Supplementary material for: Palliative care in the Eastern Mediterranean: comparative analysis using specific indicators
Source: BMC Palliat Care. 2022 Oct 3;21:168. doi: 10.1186/s12904-022-01047-7 (PMC9528121; doi:10.1186/s12904-022-01047-7)
Supplement: Supplementary file 1 — Additional file 1. [file 12904_2022_1047_MOESM1_ESM.pdf]

PALLIATIVE CARE DEVELOPMENT STATUS IN THE EMRO REGION
UPDATE 2020

Dear Participant,

This project focuses on assessing the current status and future development of Palliative Care in the Eastern Mediterranean Region.This is an initiative of the author institute 1 through its author institution 2, commissioned to the Regional Network of Experts of Palliative Care in the EMRO region, and the author institution 3. The aim of the project is to monitor and evaluate the implementation of the regional palliative care development.

In this part of the project we are inviting you to answer the questions of this survey in order to provide the data on the development of palliative care in your country. These questions have been developed by the author institution 3 based on comments of EMRO experts in the previous consensus rounds.

The survey is divided in four WHO domains: policy, education, use of medicines, service provision; and one extra domain: professional activity. Every domain contains the indicators that succesfuly passed the evaluation phase of the project and a concept defining the indicator. For each indicator you will be asked several questions. At the end of each question there will be a comment box for possible comments. There will also be a comment box at the end of the survey.

Thank you for your participation,

PALLIATIVE CARE DEVELOPMENT STATUS IN THE EMRO REGION
UPDATE 2020

Your participation in this survey is voluntary. If you do not wish your name and organisation to be made public and acknowledged in the final report, there will be an option allowing you to do so below.

We are including identifiers in the final database that will be able to connect your details to your answers. This will be accessible through a password-protected document available only to the author institution 3 and the author institution 2. Once all the data have been analysed, your original questionnaire will be destroyed. We believe there is minimal risk in undertaking this survey.

You have the right to access your personal data, modify them, cancel them or decline public acknowledgement if you wish, as well as to exercise other rights that are listed on the Data Protection Page, where you can obtain more details about the author institution 3 privacy policy. If you wish to clarify anything or exercise your rights, please contact XXXX. You may withdraw from the study at any time.

Information from this survey will be used to prepare a technical report and a further survey on the current status of palliative care progress in the region. By continuing with the survey, you agree to take part in this study.

You can exit the survey at any time and your responses will be saved once you have finished each section. Please rate the indicators to the best of your knowledge.

You can also download the questionnaire in a pdf format, so that you can read the questions before you complete it or by using the pdf form and returning it to us via e-mail if you prefer.

You will be formally acknowledged in the acknowledgement section of the technical report. If you do not wish to be publicly acknowledged please tick the box below.

☐ I do not want to be publicly acknowledged

* **Contact information (your name and affiliation will not be publicly acknowledged if you have ticked in the box above)**

Name (as you would like it to appear in the acknowledgements) ____________

Affiliation (as you would like it to appear in the acknowledgements) ____________

Country ____________

Policy domain

INDICATOR 1: Existence of a current national palliative care plan, programme, policy or strategy

Concept: National plan or programme refers to regulatory and official publications that are applicable to the whole country (these could be in the form of laws or other official documents). These publications are usually endorsed by the national health authority and contain norms and standards for the development of palliative care, Regulations relating to its service provision and in some cases guidelines for palliative care research. It should: (I) be of national scope; (II) be designed to integrate palliative care in health care services; (III) have an allocated budget; and (IV) have an assigned focal point at national level.

Does your country have any of the following? Please select

	Yes	No	In progress	Don't know
A reference to palliative care in national strategies for cancer	◯	◯	◯	◯
A reference to palliative care in national strategies for non-communicable diseases (e.g AIDS or cardiovascular disease)	◯	◯	◯	◯
A national strategy or plan specific to palliative care	◯	◯	◯	◯
A national palliative care strategy that has been implemented and is regularly evaluated	◯	◯	◯	◯
A national palliative care strategy that has been implemented and is regularly evaluated and updated	◯	◯	◯	◯
A designated branch/unit/desk/person within the Ministry of Health with responsibility for palliative care	◯	◯	◯	◯

Comment

INDICATOR 2: Existence of a specific palliative care national law

Concept: This indicator measures the existence of national legislation specific to palliative care.

Does your country have any of the following? Please select.

	Yes	No	In progress	Don't know
A reference to palliative care in decrees / norms other than in a national law (could be a regional law)	◯	◯	◯	◯
A reference to palliative care in a national law	◯	◯	◯	◯
A specific (stand-alone) palliative care law or recognition of palliative care as a right in the constitution	◯	◯	◯	◯
Do-not-resuscitate(DNR)-related laws and regulations	◯	◯	◯	◯

Comment

INDICATOR 3: Inclusion of palliative care services in the basic package of health services

Concept: Basic package of health services are a set of services identified by countries which stipulates what should be available and provided at primary health care level in the country. Based on global and regional UHC commitments, one of those services included in the basic package of health services should be palliative care. This indicator aims at assessing only the inclusion of palliative care in the list of services provided at primary care level but not its implementation. The inclusion of the specific palliative care term in the list is compulsory in order to answer “yes” to this indicator.

Have palliative care services been included in the basic package of health services? Please select.

- ◯ Yes, for all patients, with any diseases
- ◯ Yes, but only for patients with oncological diseases
- ◯ No
- ◯ I do not know

Comment

INDICATOR 4: Allocation of funds for palliative care in the national health budget by the Ministry of Health or equivalent Government Agency

Concept: Ministry of Health or equivalent government agency has reserved some type of funding in the annual national health budget for palliative care provision.

How is palliative care funded in your country? Please select all applicable options.

- ☐ Government-funded on a regular basis
- ☐ Partial government funds (pilot projects)
- ☐ Health insurance or national pension schemes
- ☐ Costs met by non-profit organisations (e.g. NGOs, charities, civil society)
- ☐ Direct payment by patients / their relatives
- ☐ Other (please specify)

INDICATOR 5: Inclusion of palliative care in private health insurance

Concept: Insurance companies consider palliative care as subject of the range of conditions to be covered by their insurance plan.

Is palliative care included in health insurance plans? Please select

- ☐ Don't know
- ☐ Not covered by health insurance
- ☐ Yes, but it is covered only at certain moments (e.g. end of life)
- ☐ Yes, but it is covered only for some patients (e.g. cancer)
- ☐ Yes, but coverage is limited by type of insurance
- ☐ Coverage is available to all patients

Comment

PALLIATIVE CARE DEVELOPMENT STATUS IN THE EMRO REGION
UPDATE 2020

Services domain

INDICATOR 6: Number of specialized palliative care services in the country per population

Concept: For the purpose of this questionnaire we are interested in the total number of palliative care services operating in your country. These include, but are not limited to: (I) free standing hospices with or without inpatient beds, (II) hospices that are a part of public or NGO hospitals, (III) home care teams, (IV) palliative care support teams in hospitals, (V) palliative care inpatient and outpatient facilities, (VI) pediatric palliative care hospices and services. Our focus is on services that are providing specialized / specialist palliative care as their primary mission. We also note that a palliative care service provider organization may have more than one local service in operation. Please therefore report information on the number of palliative care services in your country, recognizing that this is likely to be greater than the number of provider organizations. Importantly, Palliative Care services may be unequally distributed in the country causing accessibility problems and overall big in-country differences between regions, cities, rural and urban areas, etcetera. We want to measure to what extent the different modalities of provision of palliative care services across the country extend geographically.

What was the total number of palliative care services operating in your country in 2020?

Is the above figure your personal estimate?

- Yes
- No - please give the source of the figure

(Optionally) Please upload a file of the source or send it to us via e-mail with the title 'source number of PC services'

Source-file of the figure:

Choose File Choose File No file chosen

Comment

In your opinion, are the following types of palliative care services available in your country?

	Yes	No	In progress	Don't know
Home palliative care teams	○	○	○	○
Inpatient palliative care units in hospitals (public and private)	○	○	○	○
Specialized hospital palliative care support teams	○	○	○	○
Inpatient hospices	○	○	○	○
Outpatient facilities	○	○	○	○

Comment

In your opinion, what is the geographical spread of the following types of palliative care services in your country?

	1 (restricted to very few locations)	2	3	4	5	6	7	8	9	10 (widely available)
Home palliative care teams	○	○	○	○	○	○	○	○	○	○
Inpatient palliative care units in hospitals (public and private)	○	○	○	○	○	○	○	○	○	○
Specialized hospital palliative care support teams	○	○	○	○	○	○	○	○	○	○
Inpatient hospices	○	○	○	○	○	○	○	○	○	○
Outpatient facilities	○	○	○	○	○	○	○	○	○	○

Comment

INDICATOR 7: Children's palliative care provision

Concept: This indicator explores the palliative care provision for children in your country.

Which of the following categories best describes palliative care activity related to children in your country?

- ○ No known palliative care activity for children.
- ○ Capacity building palliative care activity for children.
- ○ Isolated children's palliative care provision.
- ○ Generalised children's palliative care provision.
- ○ Children's palliative care services are available through a variety of palliative care providers and types of services.
- ○ Children's palliative care services are available through a variety of palliative care providers and types of services on an continuos basis.

Comment

INDICATOR 8: Availability of centres of excellence for palliative clinical care, education and research

Concept: A center of excellence for palliative care is a program within a healthcare institution which is assembled to supply an exceptionally high concentration of expertise and related resources centered on palliative care. It delivers associated care in a comprehensive, interdisciplinary fashion to afford the best patient outcomes possible. Palliative care is delivered in a unique and focused manner to patients. Besides the center has significant research component and the staff members receive clinical capacity building to ensure that they understand and can relate to patients (adapted from Elrod and Fortenberry, 2017).

Are there centres of excellence for palliative clinical care, education and research available in your country?

- ◯ Yes, there are centres in every region
- ◯ Yes, available in more than half the regions
- ◯ Yes, available in less than half the regions
- ◯ Yes, there is one centre of excellence
- ◯ In progress
- ◯ No
- ◯ Don't know

Comment

Pain medicines domain

INDICATOR 9: Availability of morphine and other strong opioids

Concept: This indicator measures whether or not the country has immediate-release morphine generally available in primary health care facilities in the public health sector.

Does your country have access to the following drugs? Select access.

	Always	Usually	Occasionally	Never	Don't know
Injectable morphine	○	○	○	○	○
Oral morphine	○	○	○	○	○

Comment

Please tell us to what extent patients in need of palliative care in your country have access to each step of the ladder. Select access.

	Always	Usually	Occasionally	Never	Don't know
Step 1 of the ladder (e.g. paracetamol, aspirin)	○	○	○	○	○
Step 2 of the ladder (e.g. codeine, tramadol)	○	○	○	○	○
Step 3 of the ladder (e.g. morphine, fentanyl)	○	○	○	○	○

Comment

Education domain

INDICATOR 10: Existence of a process of official specialisation in Palliative Medicine for physicians, recognised by the competent authority

Concept: Official specialisation in palliative medicine refers to any formal process (or schedule of training/education) that provides official certification and accredits a higher level of competence to the physician working in the area of palliative medicine.

Existence of an official process for the specialization in palliative medicine for physicians, accredited by the national responsible authority (as specialty, sub-specialty, special area of competence or other advanced accreditation diploma)? Please select.

- ○ Yes, specialty
- ○ Yes, sub-speciality.
- ○ Yes, special area of competence or other advanced accreditation diploma.
- ○ No, but a process of specialisation is in progress.
- ○ No, but a specialisation done abroad is officially recognized in the country.
- ○ No, but basic training courses are available in the country
- ○ No, but informal process of training is available in the country
- ○ No, there is no established, in progress, or, recognised specialisation process or diplomas to certify competency
- ○ Don't know

In case you have a specialization, please estimate the number of certified palliative care professionals.

Comment

INDICATOR 11: Education for pre-qualification doctors/nurses

Concept: This indicator explores palliative care undergraduate education and training in the country.

Please fill in the next questions about medical schools

What is the total number of medical schools in the country?

Number of medical schools that offer a mandatory course or subject specifically dedicated to palliative care as part of their curricula.

Number of medical schools that offer mandatory palliative care education in combination with other related disciplines (e.g. a mandatory course of oncology and palliative care).

Number of medical schools that teach mandatory palliative care transversally, integrated into the curricula.

Number of medical schools that teach palliative care optionally.

Please fill in the next questions about nursing schools

What is the total number of nursing schools in the country?

Number of nursing schools that offer a mandatory course or subject specifically dedicated to palliative care as part of their curricula.

Numer of nursing schools that offer mandatory palliative care education in combination with other related disciplines (e.g. a mandatory course of oncology and palliative care).

Number of nursing schools that teach mandatory palliative care transversally, integrated into the the curricula

Number of nursing schools that teach palliative care optionally

Palliative care teaching is not available at universities but in other institutions. Please select.

- [ ] Non-profit sector
- [ ] Hospices organisations
- [ ] Primary care sector
- [ ] University Hospital
- [ ] Medical Centres
- [ ] Ministry of Health
- [ ] Other
- [ ] Comment

Professional activity domain

INDICATOR 12: Existence of professional vitality regarding palliative care

Concept: This indicator explores aspects regarding professional vitality in palliative care, such as the existence of at least one national palliative care association, the existence of a palliative care services directory, a national journal of palliative care, and a palliative care congress.

Does your country have any of the following? Please select.

	Yes	No	In progress	Don't know
Evidence of palliative care professional or policymeetings	◯	◯	◯	◯
Existence of a palliative care national association	◯	◯	◯	◯
A directory of palliative care services that is updated regularly	◯	◯	◯	◯
Palliative care clinical standards or clinical guidelines	◯	◯	◯	◯
A national journal of palliative care	◯	◯	◯	◯
A national palliative care conference once every year or once every two years	◯	◯	◯	◯
Evidence of professional co-operation with other specialties outside palliative care	◯	◯	◯	◯

Comment

INDICATOR 13: Existence of grants to finance palliative care research

Concept: This refers to the existence of designated amounts of money to specifically fund palliative care research, completely or partially. Sources can be a government, universities, foundations, national or international (health) associations, etcetera.

Please answer the following scale

	Many	Some	Almost none	None	Don't know
Are there grants in your country for the financing of palliative care research?	◯	◯	◯	◯	◯

INDICATOR 14: Public awareness of palliative care

Concept: The public awareness is the general society's consciousness of the need for and benefits of palliative care for patients at any time during an illness experience (adpated from Stejnsward, 2007)

What is the level of public awareness of palliative care in your country?

- ◯ Don't know
- ◯ There is no type of social awareness for palliative care
- ◯ Most people don't care about palliative care
- ◯ Some sectors of the society recognize the importance of palliative care
- ◯ A significant proportion of the society agrees with the importance of palliative care
- ◯ The public awareness of palliative care is generalised in the society

Please comment on what facts are you basing your choice of category (e.g. presence of palliative care in media)

PALLIATIVE CARE DEVELOPMENT STATUS IN THE EMRO REGION
UPDATE 2020

This is the end of the survey

Thank you very much for your time!

Please feel free to make any final comments here about the survey.
