# Supplementary material for: Palliative care in the Eastern Mediterranean: comparative analysis using specific indicators
Source: BMC Palliat Care. 2022 Oct 3;21:168. doi: 10.1186/s12904-022-01047-7 (PMC9528121; doi:10.1186/s12904-022-01047-7)
Supplement: Supplementary file 2 — Additional file 2. [file 12904_2022_1047_MOESM2_ESM.docx]

| Dimension | Country | Iran | Morocco | Oman | Egypt | Jordan | Qatar | Lebanon | Kuwait | Saudi Arabia | Iraq | Pakistan | Palestine |
| --- | --- | --- | --- | --- | --- | --- | --- | --- | --- | --- | --- | --- | --- |
| Politics | A national strategy plan | 1 | 1 | 0 | 1 | 1 | 1 | 1 | 1 | 1 | 0 | 1 | 0 |
|  | Palliative care law | 0 | 0 | 0 | 0 | 0 | 1 | 0 | N | 0 | 0 | 0 | 0 |
|  | DNR-related laws and regulations | 0 | 0 | 1 | 0 | 1 | 1 | 1 | 0 | 1 | 0 | 0 | 0 |
|  | PC included basic package | 0 | 0 | 1 | 0 | 1 | 1 | 1 | 1 | 1 | 0 | 0 | 0 |
|  | Funds for palliative care | 0 | 0 | 1 | 1 | 0 | 1 | 1 | 1 | 1 | 1 | 0 | 0 |
|  | Private health insurance | 0 | 0 | 1 | N | 1 | 1 | 1 | N | 1 | 1 | 1 | 0 |
| Education | Specialization in PC | 1 | 0 | 0 | 0 | 1 | 1 | 1 | 0 | 1 | 0 | 1 | 0 |
|  | Medical schools with mandatory course | 0 | 1 | 1 | 0 | 0 | 1 | 1 | 1 | 0 | 0 | 0 | 1 |
|  | Nursing schools with mandatory course | 0 | 0 | 1 | 0 | 1 | 1 | 1 | 1 | 0 | 0 | 0 | 0 |
| Medical | Availability of Injectable morphine | 1 | 0 | 1 | 0 | 1 | 1 | 0 | 1 | 1 | 0 | 0 | 1 |
|  | Availability of Oral morphine | N | 1 | 1 | 0 | 1 | 1 | 1 | 1 | 1 | 0 | 0 | 1 |
|  | Consumption opiods (OME) 2017 | 0 | 0 | 0 | 0 | 0 | 1 | 1 | 1 | 1 | N | N | N |
| Services | PC services per 100,000 people | 0 | 0 | 0 | 1 | 0 | 1 | 1 | 1 | 1 | 0 | 0 | N |
|  | Available Home palliative care teams | 1 | 1 | 1 | 1 | 1 | 0 | 1 | 1 | 1 | 0 | 1 | 0 |
|  | Available Inpatient palliative care units in hospitals (public and private) | 1 | 1 | 1 | 1 | 1 | 1 | 1 | 1 | 1 | 0 | 1 | 0 |
|  | Available Specialized hospital palliative care support teams | 0 | 1 | 1 | 1 | 1 | 1 | 1 | 1 | 1 | 1 | 1 | 0 |
|  | Available Inpatient hospices | 0 | 0 | 0 | 1 | 1 | 0 | 0 | 1 | 1 | 0 | 1 | 0 |
|  | Available Outpatient facilities | 0 | 1 | 1 | 1 | 1 | 1 | 1 | 1 | 1 | 0 | 1 | 0 |
|  | Geographical spread (median value) | 0 | 1 | 0 | 0 | 1 | 1 | 0 | 0 | 1 | 0 | 1 | N |
|  | Children´s palliative care provision | 0 | 1 | 0 | 1 | 1 | 1 | 1 | 1 | 1 | 0 | 1 | 1 |
| Vitality | Centres of excellence for PC | 0 | 1 | 1 | 0 | 1 | 1 | 0 | N | 1 | 0 | 1 | 0 |
|  | Professional or policymeetings | 1 | 0 | 0 | 0 | 1 | 1 | 1 | 1 | 1 | 0 | 0 | 0 |
|  | Palliative care national association | 0 | 1 | 1 | 0 | 1 | 0 | 1 | 1 | 1 | 0 | 0 | 0 |
|  | Directory of palliative care | 0 | 1 | 0 | 0 | 0 | 1 | 0 | 1 | 1 | 0 | 0 | 0 |
|  | Palliative care clinical standards | 0 | 1 | 0 | 0 | 1 | 1 | 0 | 1 | 1 | 0 | 0 | 0 |
|  | National journal of PC | 0 | 0 | 0 | 0 | 0 | 0 | 0 | 0 | 1 | 0 | 0 | 0 |
|  | National palliative care conference | 1 | 0 | 0 | 0 | 1 | 0 | 1 | 0 | 0 | 0 | 1 | 0 |
|  | Professional co-operation | 1 | 1 | 0 | 0 | 1 | 1 | 1 | 1 | 0 | 1 | 1 | 0 |
|  | Grants to finance PC research | 1 | 1 | 0 | 0 | 1 | 1 | 0 | N | 1 | 0 | 0 | 0 |
|  | Public awareness of PC | 0 | 1 | 1 | 0 | 0 | 1 | 0 | 0 | 0 | 0 | 0 | 0 |
